# Dietary Defective Jujube as a Corn Substitute: Impacts on Growth Performance, Meat Traits, and *Alternaria* Toxin Exposure in Lambs

**DOI:** 10.3390/ani16020255

**Published:** 2026-01-14

**Authors:** Letian Zhang, Haoyang Hui, Muhammad Faheem, Yanfeng Xue, Ning Chen, Xiaoling Zhou

**Affiliations:** 1 College of Animal Science and Technology, Tarim University, Alar 843300, China; tdzlt2523@163.com (L.Z.); 18299524164@163.com (H.H.); 2 Key Laboratory of Livestock and Forage Resources Utilization Around Tarim, Ministry of Agriculture and Rural Affairs, Tarim University, Alar 843300, China; 3 Department of Clinical Sciences, Faculty of Veterinary Science, Bahauddin Zakariya University, Multan 60800, Pakistan; faheem_dear@hotmail.com; 4 College of Animal Science and Technology, Anhui Agricultural University, Hefei 230000, China; xueyanfeng1990@163.com; 5 State Key Laboratory of Sheep Genetic Improvement and Healthy Production, Xinjiang Academy of Agricultural and Reclamation Sciences, Shihezi 832000, China

**Keywords:** jujube by-products, lamb, growth performance, blood biochemical indices, meat quality, *Alternaria* toxin

## Abstract

This study evaluated the effects of replacing corn with defective jujube (DJ) on growth, digestibility, blood biochemical indices, meat performance, and the presence of *Alternaria* toxin residues in Karakul lambs. Thirty-six lambs were split into groups given 0%, 15%, or 30% DJ, replacing 0%, 45.45%, and 90.91% of corn. The trial lasted 75 days, with 15 days for adaptation and 60 days for measurement. Digestibility for crude protein and ether extract of male lambs increased in the DJ30 group over CON (*p* < 0.05). High-density lipoprotein decreased in DJ30 (*p* < 0.01), while triglycerides and total cholesterol in DJ30 dropped (*p* < 0.05). Blood urea nitrogen and aspartate aminotransferase decreased in DJ15 and DJ30 (*p* < 0.01). Superoxide dismutase and catalase rose in DJ30 (*p* < 0.01), while malondialdehyde declined (*p* < 0.05). Growth hormone and insulin-like growth factor-1 increased in DJ30 (*p* < 0.01). Feeding DJ did not affect meat production or quality. No *Alternaria* toxins were detected in rumen, liver, or meat. Feeding 15–30% DJ improved nitrogen utilization, lipid metabolism, and blood antioxidant levels in lambs and reduced the risk of liver damage, while no *Alternaria* toxin remained in organs. A 30% DJ substitution for corn is a safe strategy for lamb feeding.

## Simple Summary

Using by-products such as defective jujube in ruminant feed helps address global feed shortages and lowers costs. Defective jujube shares nutritional traits with corn and contains bioactive compounds, including flavonoids, polyphenols, and vitamin C. This study evaluated whether defective jujube could replace corn as an energy feed. Thirty-six Karakul lambs (3 months old, 19.43 ± 2.55 kg) were randomly assigned to three groups and fed 0% (CON), 15% (DJ15), or 30% (DJ30) DJ. Results showed that defective jujube substitution sustained normal growth, enhanced blood antioxidant capacity, improved nutrient utilization efficiency, and regulated blood lipid metabolism in Karakul lambs. Defective jujube can be substituted for corn, and up to 30% replacement in the Karakul lamb diet is safe.

## 1. Introduction

Chinese jujube (*Ziziphus jujuba* Mill.), one member of the Rhamnaceae family, has been cultivated in China for 4000 years [1,2]. Approximately 7 million tons of jujube are produced each year in China, accounting for over 90% of global production [3]. Roughly 30% of these fruits are discarded each year due to physical damage, odd or undersized shapes that do not meet commercial standards [3,4]. These are called defective jujube (DJ). DJ contains 4.5–7.7% crude protein (CP), 0.27–1.15% ether extract (EE), and 12–15 MJ/kg of digestible energy (DE) [5]. It also contains bioactive compounds (Table 1), which are important for growth and antioxidant activity [5,6]. The cost of DJ is ¥500–700 per ton, lower than that of corn, and it is readily available, making it a good feed option for livestock.

The feeding value of jujube and its by-products has been shown in monogastric livestock and poultry, including pigs, rabbits, and broilers [11,12,13]. In ruminants, Liu et al. [13] reported that adding 7.5% fermented jujube powder to Simmental bull diets improved growth performance, nutrient absorption, and antioxidant capacity. Xie et al. [8] and Zhang et al. [14] reported that replacing corn with jujube improved goat production performance and meat quality. We speculate that this may be because the polysaccharides, flavonoids, and other bioactive components in jujube help regulate rumen fermentation, improve nutrient digestion and utilization, and enhance the body’s antioxidant capacity. To our knowledge, limited research has been conducted on sheep, and the low inclusion levels commonly used in these studies do not fully realize the economic advantage of DJ in practical production. However, DJ is susceptible to *Alternaria fungal* contamination due to physical damage or poor storage [15]. These fungi primarily produce toxins such as tenuazonic acid (TeA), alternariol (AOH), and alternariol monomethyl ether (AME) [16,17]. These toxins are carcinogenic, genotoxic, and cytotoxic [17,18,19], introducing potential risks for the use of DJ as feed. To date, little information is available on the safety limitations of *Alternaria* toxins in livestock feed and their metabolic processes in vivo.

Karakul sheep, an indigenous breed in southern Xinjiang, China, is known for its adaptability to coarse fodder and arid desert environment, and superior meat quality. Given the nutritional quality of jujube, this study aimed to evaluate the effects of partially replacing corn with DJ in the diet on growth performance, blood biochemical parameters, meat quality, and presence of *Alternaria* toxin residues in Karakul lambs. The results aim to inform the use of DJ as an alternative energy feed in sheep production.

## 2. Materials and Methods

### 2.1. Animal Ethics

The purebred Karakul sheep used in this study were purchased from a farm in Alaer, Xinjiang, where they had been confined and raised in large groups with ewes. All sheep were singletons. All experimental procedures in this study were approved by the Animal Ethics Committee of Tarim University (approval number: PB20251202001).

### 2.2. Defective Jujube

The DJ was purchased from Hongfutian Jujube Co., Ltd. in Alaer, Xinjiang, China. It was subsequently dried, ground, and incorporated into the diet. The chemical composition of DJ is shown in Table 2.

### 2.3. Experimental Design and Materials

The experiment was conducted at the Experimental Station of the College of Animal Science and Technology at Tarim University, from May to July 2024. Thirty-six Karakul lambs (19.43 ± 2.55 kg body weight, 90 days old) were randomly allocated into three treatments (*n* = 12 per treatment) and fed diets containing different levels of DJ: CON (0%), DJ15 (15%), and DJ30 (30%), which replaced 45.45% and 90.91% of dietary corn, respectively. The lambs in each treatment group were housed in four pens, each containing three individual stalls (1.5 m × 2 m per stall) for single-lamb housing, and the plastic floor served as the sheep’s bed.

Diets were formulated according to NY/T 816-2021 [23] to support an average daily gain (ADG) of 0.2 kg/day. The diet consists of 60% concentrate and 40% roughage (cottonseed hull and rice hull). Cottonseed hull and rice hull are commonly used as feed ingredients for fattening lambs in southern Xinjiang, China. Although their physical structure differs from that of long hay, they are widely used to provide basic fiber under local feeding conditions. The experiment comprised a 15-day adaptation period followed by a 60-day formal trial. Before the adaptation period, all lambs were dewormed and screened for Brucellosis infection. Feed was provided in equal amounts twice daily at 10:00 and 19:00, with free access to water throughout the experimental period. Daily feed intake and feed refusals were recorded. The daily feed was then adjusted accordingly to ensure that feed residue did not exceed 5% of the feed offered (Table 3).

### 2.4. Sample Collection and Determination

#### 2.4.1. Growth Performance

ADG was calculated based on body weights measured on two consecutive days before morning feeding on days 0 and 40. Average daily feed intake (ADFI) and the feed-to-gain ratio (F/G) were derived from the daily feed intake records using the following formulas:

ADFI (g/d) = Total Feed Intake/Days

ADG (g/d) = (Final Body Weight − Initial Body Weight)/Days

F/G = (ADFI/ADG) × 100%

#### 2.4.2. Apparent Digestibility

On day 41, four male lambs per group were randomly selected and housed in metabolism cages for a 12-day adaptation. On days 53 to 59, fresh feces were collected from the rectum of each lamb 2 h after feeding. The samples collected each day were pooled in equal amounts, from which a 100 g subsample was taken, treated with 10 mL of 10% H_2_SO_4_ for nitrogen fixation, and subsequently stored at −20 °C. After the trial, all fecal samples from each lamb were thoroughly mixed, dried at 65 °C, and ground before analysis. Dry matter (DM), CP, EE, and other indicators in feed and feces were obtained by chemical analysis. Nutrient apparent digestibility was calculated using the hydrochloric acid-insoluble ash (AIA) method with the following formula:

$$\mathrm{Apparent}\;\mathrm{Digestibility}\;(\%)\;=\;\left[1-\frac{\left(\mathrm{Content}\;\mathrm{of}\;\mathrm{Nutrient}\;\mathrm{in}\;\mathrm{Feces}\;\times \;\mathrm{AIA}\;\mathrm{Content}\;\mathrm{in}\;\mathrm{Diet}\right)}{\left(\mathrm{Content}\;\mathrm{of}\;\mathrm{Nutrient}\;\mathrm{in}\;\mathrm{Ingested}\;\mathrm{Diet}\;\times \;\mathrm{AIA}\;\mathrm{Content}\;\mathrm{in}\;\mathrm{Feces}\right)}\right]\times 100$$

#### 2.4.3. Plasma Biochemical Indices

On days 20 and 40, jugular venous blood samples (10 mL) were drawn from each lamb before morning feeding into EDTA-coated tubes. The plasma fraction obtained by centrifugation at 1000× *g* for 10 min was analyzed for a panel of biochemical parameters. This panel included alanine aminotransaminase (ALT), aspartate aminotransaminase (AST), total protein (TP), blood urea nitrogen (BUN), glucose (GLU), triglyceride (TG), total cholesterol (TC), high-density lipoprotein (HDL), and low-density lipoprotein (LDL), which were assayed using an automated biochemical analyzer (Photometer 5010, Robert Riele GmbH & Co KG, Berlin, Germany). The activities of catalase (CAT), superoxide dismutase (SOD), and glutathione peroxidase (GSH-Px), as well as the concentrations of malondialdehyde (MDA), insulin (INS), growth hormone (GH), and insulin-like growth factor-1 (IGF-1), were determined using commercial ELISA kits (MeiMian, Jiangsu MeiMian Industrial Co., Ltd., Yancheng, China).

#### 2.4.4. Slaughter Performance

On day 60, after a 24 h fast and a 2 h water deprivation period, all lambs were humanely slaughtered. The body weights before slaughter (BWS) were recorded. After slaughter, the longissimus dorsi (LD) muscles between the 12th and 13th ribs were collected to determine LD muscle area. Hot carcass weight (HCW) and the weights of visceral organs (heart, liver, spleen, lung, kidney, pancreas) were recorded to calculate dressing percentage and organ indexes. Calculations were performed using the following formulas:

Dressing Percentage (%) = (HCW/BWS) × 100

Organ Index (%) = [Organ Weight (g)/BWS (kg)] × 100

#### 2.4.5. Meat Quality Determination

Within 30 min post-slaughter, LD muscle samples were excised from the left side of each lamb carcass. After trimming visible connective tissues and adipose deposits, pH and meat color parameters were measured. Additionally, the chemical composition of meat samples was analyzed according to AOAC [21] methods: moisture (934.01), CP (2001.11), and EE (2003.05). For fatty acid composition determination, the pretreatment was performed as described by Cao et al. [24]. The analysis of fatty acid methyl esters was performed on a gas chromatograph (7820A, Agilent Technologies, Santa Clara, CA, USA) equipped with a flame ionization detector and an SP-2560 fused silica capillary column (100 m × 0.25 mm × 0.2 μm).

LD muscle samples (3 cm × 1 cm × 1 cm) were excised after removing fascia, tendons, and adipose tissue. The samples were placed in cooking bags and heated in 80 °C water until the meat cores reached 70 °C. After cooling to 25 °C, three cylindrical cores per sample were extracted parallel to the muscle fiber orientation using a coring device. Warner-Bratzler shear force (WBSF) was measured with a tenderness analyzer (C-LM3B, Tenovo Food, Beijing, China).

The LD muscle samples (4 cm × 3 cm ×1 cm) were excised from the third and fourth lumbar vertebrae of the left carcass, weighed, and placed in cooking bags. The samples were heated in an 80 °C water bath for 30 min and then cooled to 25 °C. After blotting dry with filter papers to remove surface moisture, the samples were reweighed, and the cooked meat percentage (CMP) was calculated.

CMP (%) = (Weight after Cooking/Weight before Cooking) × 100%

#### 2.4.6. Determination of *Alternaria* Toxin Residues

The liver tissue, LD muscle, rumen fluid, and diet samples were freeze-dried at −60 °C using a freeze-dryer (FD-503, Jinan Junde Instrument Co., Ltd., Jinan, China). The lyophilized samples were ground into a powder, and the concentrations of TeA, AME, AOH, *Alternaria tenuissima* toxin (ATT), and tentoxin (TEN) were analyzed by UPLC-MS/MS according to the method described by Liu et al. [25].

### 2.5. Statistical Analysis

All experimental data were initially processed in Excel 2016. Statistical analyses were performed using IBM SPSS Statistics 27. Except for apparent digestibility data, which were analyzed by one-way ANOVA, all other data were evaluated using a general linear model with gender and treatment as fixed factors. Post hoc comparisons were performed with the Sidak test. Differences were considered statistically significant at *p* < 0.05. Data are presented as “Mean ± SD”.

## 3. Results

### 3.1. Effect of DJ on Growth Performance and Apparent Digestibility of Karakul Lambs

As shown in Table 4, initial body weight did not differ among the CON, DJ15, and DJ30 groups (*p* > 0.05). There were no differences in body weight, ADG, ADFI, and F/G throughout the trial period for the DJ15 and DJ30 groups (*p* > 0.05). These results indicate that dietary supplementation with 15% or 30% DJ did not affect normal growth in Karakul lambs. The apparent digestibility of CP and EE of male lambs in the DJ30 group was higher than that in the CON group (*p* < 0.05). The apparent digestibility of DM in male lambs was also greater in the DJ30 group compared with the CON group (*p* < 0.01). With increasing dietary replacement by DJ, the apparent digestibility of ADF of male lambs progressively decreased in the DJ15 and DJ30 groups (*p* < 0.01). In contrast, no differences in male lambs were observed among groups for the digestibility of NDF (*p* > 0.05).

### 3.2. Effects of DJ on Blood Biochemical Indices

#### 3.2.1. Blood Metabolism

**Figure 1:**
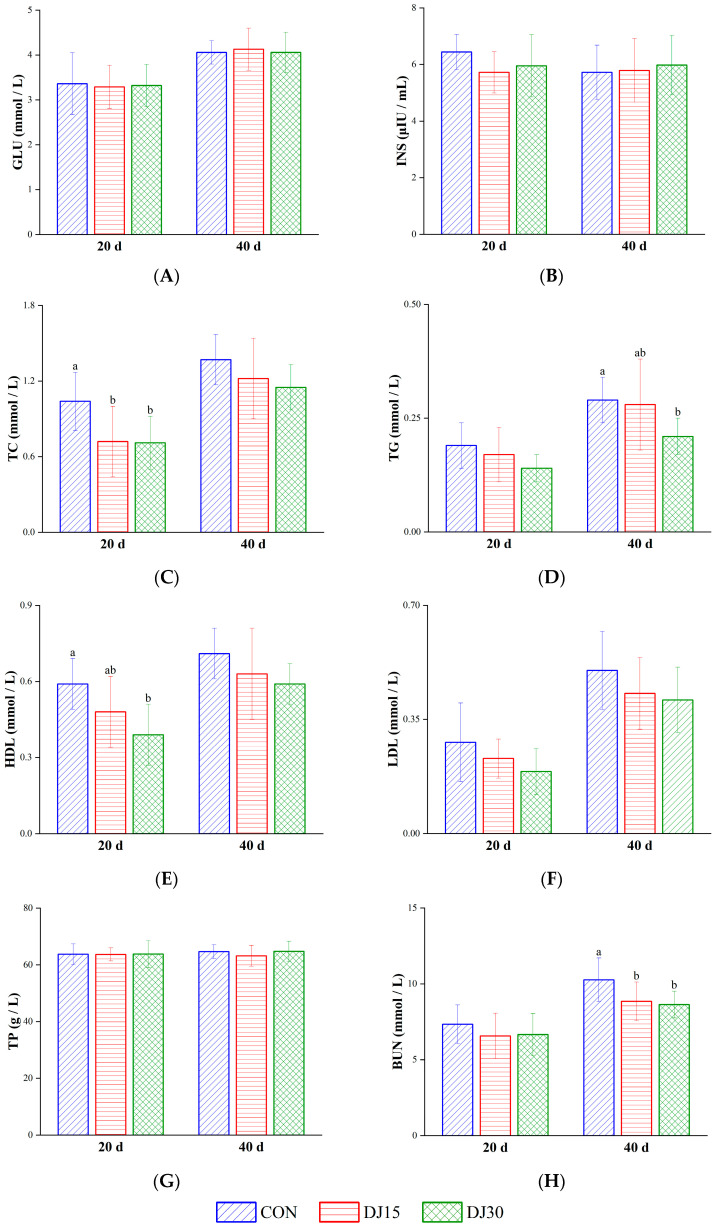
The concentrations of glucose (GLU, (**A**)), insulin (INS, (**B**)), total cholesterol (TC, (**C**)), triglyceride (TG, (**D**)), high-density lipoprotein (HDL, (**E**)), low-density lipoprotein (LDL, (**F**)), total protein (TP, (**G**)), and blood urea nitrogen (BUN, (**H**)) in the blood of different groups, respectively. Within the same sampling time point, distinct lowercase letter superscripts (e.g., a, b) indicate significant differences (*p* < 0.05).

As shown in Figure 1A,B, the concentrations of INS and GLU showed no differences on days 20 and 40 (*p* > 0.05). On day 20, the concentration of TC in the blood of the DJ15 and DJ30 groups decreased compared with that in the CON group (*p* < 0.01, Figure 1C), and the HDL concentration in the DJ30 group was also lower than that in the CON group (*p* < 0.01, Figure 1E). On day 40, the TG concentration in the DJ30 group was lower than that in the CON group (*p* < 0.05, Figure 1D). The TP and BUN concentrations did not differ among groups on day 20 (*p* > 0.05, Figure 1G,H). On day 40, the BUN concentration in the DJ15 and DJ30 groups decreased compared with that in the CON group (*p* < 0.01). The concentrations of TP and LDL (Figure 1F) were not different among groups (*p* > 0.05). Gender did not affect blood metabolism indices (*p* > 0.05).

#### 3.2.2. Antioxidant Indices

**Figure 2:**
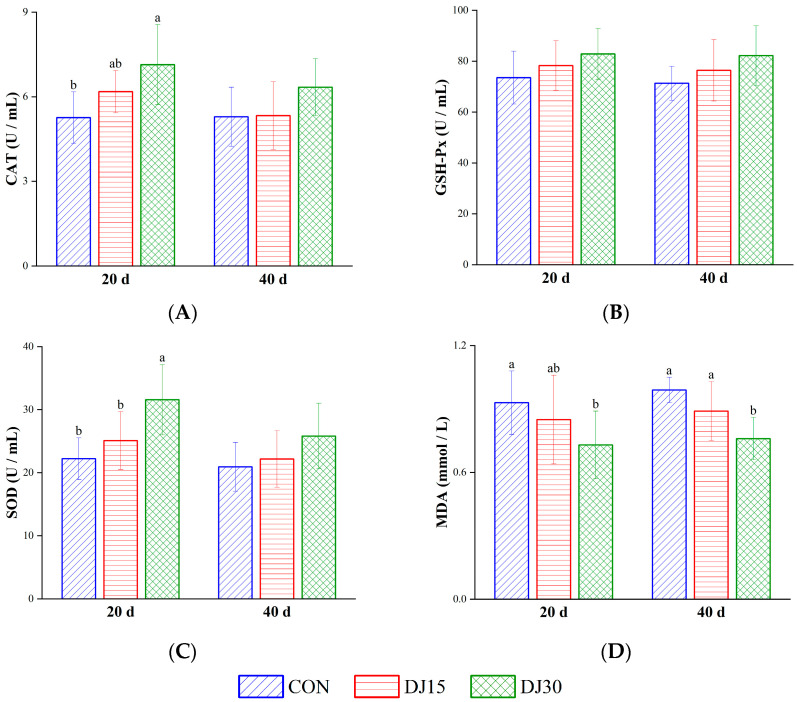
The concentrations of catalase (CAT, (**A**)), glutathione peroxidase (GSH-Px, (**B**)), superoxide dismutase (SOD, (**C**)), and malondialdehyde (MDA, (**D**)) in the blood of different treatment groups, respectively. Within the same sampling time point, distinct lowercase letter superscripts (e.g., a, b) indicate significant differences (*p* < 0.05).

On day 20, serum CAT and SOD were higher in the DJ30 group than in the CON group (*p* < 0.01, Figure 2A,C). Throughout the trial period, MDA levels were elevated in the DJ30 group compared with the CON group (*p* < 0.05, Figure 2D). No difference was observed in the activity of GSH-Px among the three groups (*p* > 0.05, Figure 2B). These results indicated that replacing corn in the diet with DJ can improve blood antioxidant capacity in lambs. Gender did not affect antioxidant indices (*p* > 0.05).

#### 3.2.3. Hepatic Function

**Figure 3:**
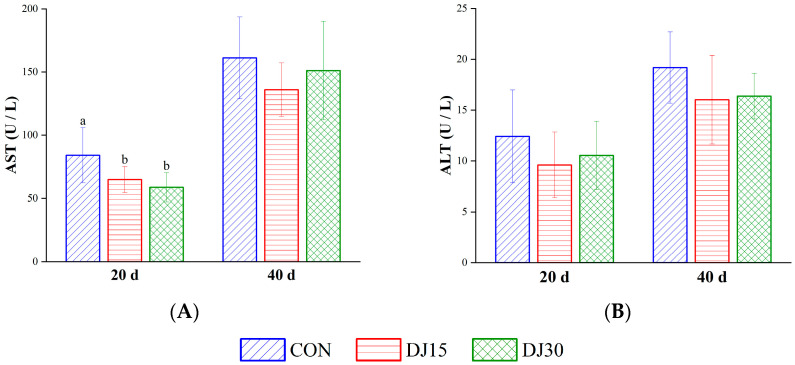
The concentrations of aspartate aminotransaminase (AST, (**A**)) and alanine aminotransaminase (ALT, (**B**)) in the blood of different treatment groups. Within the same sampling time point, distinct lowercase letter superscripts (e.g., a, b) indicate significant differences (*p* < 0.05).

As shown in Figure 3A, on day 20, AST activity decreased in both the DJ15 and DJ30 groups compared with the CON group (*p* < 0.01), whereas ALT activity was not different across groups (Figure 3B). On day 40, neither AST nor ALT activities showed intergroup variations (*p* > 0.05). Gender did not affect AST and ALT (*p* > 0.05).

#### 3.2.4. Growth-Related Hormone Indices

**Figure 4:**
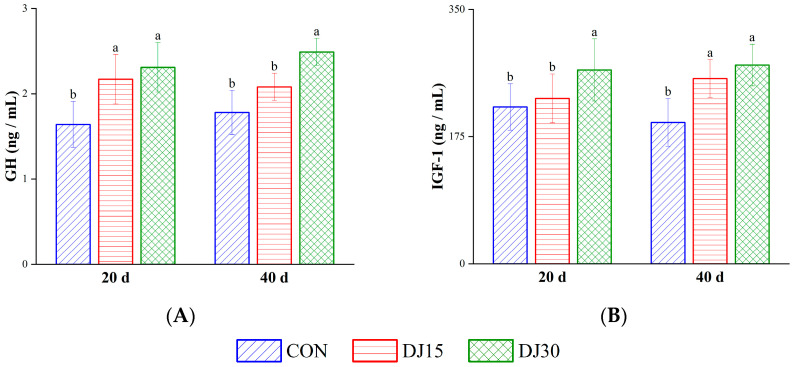
The concentrations of growth hormone (GH, (**A**)) and insulin-like growth factor-1 (IGF-1, (**B**)) in the blood of the different treatment groups. Within the same sampling time point, distinct lowercase letter superscripts (e.g., a, b) indicate significant differences (*p* < 0.05).

On day 20, the concentration of GH in both the DJ15 and DJ30 groups increased compared with that in the CON group (*p* < 0.01, Figure 4A). The concentration of IGF-1 in the DJ30 group was also higher than that in the CON group (*p* < 0.01, Figure 4B). On day 40, the GH concentration in the DJ30 group increased compared with that in the CON group (*p* < 0.01). And the concentration of IGF-1 in both the DJ15 and DJ30 groups was higher than that in the CON group (*p* < 0.01). Gender did not affect GH and IGF-1 (*p* > 0.05).

### 3.3. Effects of DJ on Slaughter Performance and Organ Indices

As presented in Table 5, no differences (*p* > 0.05) were observed among groups in BWS, HCW, dressing percentage, or LD muscle area. Although the LD muscle areas in the DJ15 and DJ30 groups were numerically 13.16% and 19.19% larger than in the CON group, respectively, these differences were not statistically significant (*p* > 0.05). Heart index, liver index, spleen index, lung index, kidney index, pancreas index, abdominal fat index, and mesenteric fat index were similar across all these groups (*p* > 0.05). Combined with the growth performance presented in Table 4, these findings demonstrated that supplying DJ in the diet did not negatively affect growth, slaughter, or meat production performance of Karakul lambs. Gender did not affect these indices (*p* > 0.05).

### 3.4. Effects of DJ on the LD Muscle

#### 3.4.1. Meat Quality, Meat Color, and pH Value

As shown in Table 6, the physicochemical properties of the LD muscle exhibited no intergroup differences (*p* > 0.05). Gender did not affect meat quality indices (*p* > 0.05).

#### 3.4.2. Muscle Chemical Composition and Fatty Acid Profiles

As shown in Table 7, the composition of the LD muscle showed no significant intergroup differences (*p* > 0.05). Gender did not affect the chemical composition in meat (*p* > 0.05).

Table 8 shows the composition of fatty acids in LD muscle. The content of C17:0 was higher in the DJ30 groups than in the CON group (*p* < 0.01). No significant variations were detected in other measured fatty acids (FAs) among groups (*p* > 0.05). Gender did not affect the fatty acids in meat (*p* > 0.05).

### 3.5. Alternaria Toxin Concentrations

As indicated in Table 9, TeA in the diets of three groups and AME in the diets of the DJ15 and DJ30 groups were detected, with their concentrations increasing linearly in the DJ15 and DJ30 groups. However, no residues (below the detection limit) of TeA, AOH, AME, ATT, and TEN were detected in the rumen fluid, liver, and LD muscle samples of all lambs.

## 4. Discussion

This study aimed to evaluate the feasibility of DJ as a feed ingredient, with a particular focus on its impact on lambs’ health and product safety. To our knowledge, this is the first study to report the effects of DJ on growth, meat performance, and *Alternaria* toxin residues in lambs.

### 4.1. Growth Performance and Apparent Digestibility

Throughout the trial, all lambs maintained normal growth performance and health status, demonstrating that supplementation with 15% to 30% DJ had no adverse effects. The digestion test in this study was only conducted on male lambs. Although a reduction in DM digestibility was observed in the DJ30 group, it did not negatively affect lamb growth. We speculate that the decrease in DM digestibility in the DJ30 group may be associated with the relatively higher ADF content in DJ. Dietary energy and protein form the nutritional foundation for animal growth and health, and also serve as fundamental criteria for assessing feed quality. Under isoenergetic and isonitrogenous conditions, Xie et al. [8] observed increased ADG and dry matter intake (DMI), along with reduced F/G in goats when replacing 20% dietary corn with jujube powder. In this study, compared with the CON group, the DJ15 and DJ30 groups showed reductions in dietary digestible energy of 1.81% and 3.61%, and in CP content of 1.88% and 3.75%, respectively. Nevertheless, the lamb’s body weight, ADFI, ADG, and F/G were unaffected. Moreover, the apparent digestibility of CP and EE in the DJ30 group increased, and the BUN level decreased, with no change in TP. These results suggest that feeding DJ may enhance nitrogen retention and improve the utilization efficiency of EE to compensate for the effect of lower dietary energy and protein levels, and this regulatory mechanism needs to be further investigated.

Interestingly, elevated concentrations of GH and IGF-1 were observed in the DJ15 and DJ30 groups. GH is secreted by the anterior pituitary gland, circulated through the bloodstream to the liver, and stimulates the synthesis and release of IGF-1 [26,27]. GH and IGF-1 play synergistic roles in promoting growth and development, regulating energy expenditure, and maintaining blood glucose homeostasis [28,29,30]. Previous studies reported that under positive energy balance, GH, IGF-1, and INS act synergistically to promote nitrogen retention; conversely, during negative energy balance, GH stimulates lipolysis [31,32]. Additionally, IGF-1 can downregulate the expression of genes associated with protein degradation [30]. Although dietary CP and energy levels were reduced by replacing corn with DJ, the improved apparent digestibility of CP and EE, coupled with increased serum GH and IGF-1 levels, enabled Karakul lambs to maintain normal growth performance. We speculate that the bioactive components in DJ initiate a compensatory effect by modulating growth-related hormone secretion and enhancing digestibility, thereby improving nutrient utilization efficiency to offset dietary nutritional limitations. However, it remains unclear how this nutritional compensatory effect acts, either by promoting nitrogen retention or by regulating fat metabolism and deposition. The specific pathways and regulatory targets involved require further investigation.

### 4.2. Metabolism of Carbohydrates and Lipids in the Blood

The mature jujubes contain high levels of soluble sugars, including glucose, fructose, and sucrose. Excessive intake of soluble sugars may disrupt glucose homeostasis and reduce INS sensitivity, which in turn can disturb lipid metabolism and promote fat deposition [33]. However, GLU and INS concentrations remained unaffected in our trial, consistent with the finding in goats fed jujube meal [34]. Our findings demonstrated that replacing corn with DJ in the diet was a safe dietary strategy that did not cause hyperglycemia in Karakul lambs.

On day 20, the concentration of TC in the blood of the DJ15 and DJ30 groups was higher than that of the CON group, and the concentration of HDL in the DJ30 group was lower than that in the CON group. On day 40, the TG concentration in the DJ30 group was lower than in the CON group. Previous studies have confirmed the hypolipidemic effects of jujube polysaccharides, including cholesterol-lowering properties [35,36]. Teng et al. [37] demonstrated that HDL facilitated reverse cholesterol transport (RCT), which shuttles peripheral cholesterol to the liver for catabolism. Beyond its growth-promoting effects, GH plays a regulatory role in lipid metabolism by facilitating lipid absorption and utilization [38]. We therefore postulate that this effect is associated with the lipid-lowering properties of jujube polysaccharides and GH, suggesting that jujube constituents have the potential to modulate lipid metabolism. Furthermore, replacing corn with DJ decreased starch intake and increased soluble sugar intake in lambs. This shift in carbohydrate composition likely altered metabolic responses. Della Corte et al. [39] reported that substituting starch with naturally occurring sugars and dietary fiber in foods reduced body fat and improved blood lipids, findings which are consistent with the blood glucose and lipid indices observed in our study.

### 4.3. Blood Antioxidant Capacity

In this study, feeding DJ elevated blood SOD and CAT activities and reduced MDA concentrations of lambs. These findings are consistent with previous observations reported by Liu et al. [13] in bulls and Zhang et al. [14] in goats. Phenolics and flavonoids in jujube have antioxidative activities [40], with hydroxyl groups in jujube polyphenols and phenolic moieties in flavonoids effectively scavenging free radicals [5]. Additionally, jujube is rich in vitamin C, which also acts as an antioxidant [34]. This demonstrates that corn substitution with DJ enhances blood antioxidative capacity in lambs, particularly at the 30% inclusion level.

### 4.4. Liver Function and Alternaria Toxin Infection

ALT and AST are metabolic markers for hepatocellular injury [41]. Although *Alternaria* toxins can cause liver damage and elevate serum ALT and AST levels, our results showed that on day 20, both ALT and AST activities of lambs in the DJ15 and DJ30 groups were lower than those in the CON group, and by day 40, no differences were observed among the three groups. This indicated that feeding DJ did not affect liver metabolism.

No *Alternaria* toxin residues were detected in the rumen fluid, liver, or LD muscle collected after slaughter. To date, the information on the concentration range of adverse reactions in sheep to *Alternaria* toxins has not been reported. Based on our findings, it can be inferred that *Alternaria* toxins were effectively degraded and did not accumulate in the lamb’s body. Several potential mechanisms may explain this observation: first, rumen microorganisms in ruminants can metabolize or hydrolyze various mycotoxins [42], which likely contribute to the detoxification; second, hepatic enzyme systems such as cytochrome P450 (CYP450) can convert foreign toxins into less toxic or non-toxic metabolites to some extent [43]; furthermore, phenolic compounds, flavonoids, and jujube polysaccharides present in DJ may synergistically enhance the antioxidant and anti-inflammatory activities of bulls and goats [13,14]. Thus, we speculated that the inactivation and clearance of *Alternaria* toxins in lambs likely result from the combined effects of multiple mechanisms, including ruminal microbial transformation, hepatic detoxification metabolism, and the protective roles of bioactive components in jujube. The specific mechanisms and metabolic pathways require further validation.

### 4.5. Meat Production Performance and Meat Quality

Although DJ substitution for corn reduced dietary energy and protein levels, it did not affect lamb slaughter performance or organ indices, consistent with results observed for ADG, body weight, and BWS. Thus, supplying 15–30% DJ did not compromise meat production in Karakul lambs. Moreover, in this trial, we detected no differences in pH, meat color, WBSF, or CMP among groups, confirming that DJ substitution for corn did not affect sensory quality attributes of LD muscle.

We observed that the C17:0 content in the LD muscle of the DJ30 group increased, with no differences in other FAs. Pfeuffer et al. [44] reported that C17:0 can be synthesized from propionic acid or other odd-chain SFAs (≤C15). We proposed that soluble carbohydrates in jujube promote the proliferation of Propionibacterium in rumen; the resultant propionic acid then serves as the precursor for C17:0 biosynthesis via the fatty acid biosynthesis pathway. However, the rumen microbiota of lambs was not analyzed in this study, which requires further investigation.

## 5. Conclusions

This study demonstrated that DJ could substitute for corn to support normal growth and development in Karakul lambs, enhance blood antioxidant capacity, and regulate lipid metabolism. The substitution of DJ for corn did not adversely affect the meat quality of lambs. Thus, DJ could be used as a cost-effective substitute for corn and is safe for lambs at inclusion levels up to 30%. However, this study focused mainly on the feeding effects of DJ on lambs, while the ruminal metabolism of bioactive compounds in DJ and the mechanisms underlying their antioxidant and lipid-lowering actions require further investigation. To fully understand its potential, future studies should also investigate the effects of DJ on ewes during gestation and lactation.

## Figures and Tables

**Table 1:** The content of main bioactive components in jujube (dry matter basis).

Items	Content	Reference
Total flavonoids (g QE/kg)	0.39–0.53	[7]
Total phenols (g GAE/kg)	4.21–5.24	[7]
Triterpenoids (mg/kg)	4198.51	[8]
Cyclic adenosine monophosphate (mg/kg)	480.92	[9]
Cyclic guanosine monophosphate (mg/kg)	236.39	[9]
Vitamin C (g/kg)	10.60	[10]

**Table 2:** Comparison of nutrient content between corn and DJ used in this study (dry matter basis).

Items ^1^	Defective Jujube	Corn
Crude protein (%)	5.98	8.52
Ether extract (%)	1.03	3.71
Calcium (%)	0.11	0.02
Phosphorus (%)	0.14	0.26
Neutral detergent fiber (%)	11.25	9.58
Acid detergent fiber (%)	8.51	2.86
Digestible energy (MJ/kg)	15.69	15.92
Soluble sugar (%)	64.89	6.76
Starch (%)	13.32	60.19

^1^ The contents of neutral detergent fiber (NDF) and acid detergent fiber (ADF) were measured according to the method of Van Soest et al. [20]. The remaining indicators were measured according to the Association of Official Analytical Chemists (AOAC) methods [21,22]. The digestible energy (DE) at the nutritional level was calculated based on the Nutrient Requirements of Sheep in China [23]: DE = 17.211 − 0.135 × NDF (% dry matter basis). The relevant indicators mentioned below are obtained in the same way.

**Table 3:** Diet composition and nutritional level (dry matter basis).

Items		Groups ^1^	
	CON	DJ15	DJ30
**Ingredients (%)**			
Cottonseed hull	20.00	20.00	20.00
Rice hull	20.00	20.00	20.00
Cottonseed meal	21.50	21.50	21.50
Defective jujube	0.00	15.00	30.00
Corn	33.00	18.00	3.00
Premix *	5.00	5.00	5.00
NaCl	0.30	0.30	0.30
NaHCO_3_	0.20	0.20	0.20
Total	100.00	100.00	100.00
**Nutrient levels**			
Digestible energy (MJ/kg)	11.62	11.41	11.20
Crude protein (%)	16.00	15.70	15.40
Ether extract (%)	4.88	4.49	4.10
Neutral detergent fiber (%)	30.42	30.56	30.69
Acid detergent fiber (%)	20.83	21.70	22.57
Calcium (%)	1.39	1.38	1.33
Phosphorus (%)	0.73	0.72	0.70

^1^ In the CON, DJ15 and DJ30 diets, corn was replaced by defective jujube meal at 0%, 15% and 30% (dry matter basis), respectively; * The premix included in the basal diet contained the following components per kg: vitamins A, D, and E at 150,000 IU, 34,000 IU, and 600 IU, respectively; iron at 2000 mg; copper at 400–600 mg; zinc at 1500–2000 mg; manganese at 600 mg; selenium at 20 mg; and iodine at 30 mg.

**Table 4:** Growth performance and apparent digestibility of lambs in three groups.

Items *		Treatment ^1^		*p*-Value	
	CON	DJ15	DJ30	Treatment	Gender
**Body weight (kg)**					
Initial	21.32 ± 3.26	20.13 ± 1.83	21.70 ± 3.19	0.416	0.546
Day 20	25.21 ± 3.62	24.29 ± 2.96	25.66 ± 3.13	0.600	0.994
Day 40	29.92 ± 4.15	28.79 ± 3.18	30.35 ± 3.79	0.605	0.891
**Average daily gain (ADG, g/d)**					
0–20 d	194.55 ± 51.79	207.92 ± 98.94	198.18 ± 32.35	0.868	0.203
20–40 d	235.45 ± 44.63	225.00 ± 31.69	234.09 ± 78.13	0.886	0.622
0–40 d	215.00 ± 45.50	216.46 ± 54.65	216.14 ± 50.35	0.989	0.546
**Average daily feed intake (ADFI, g/d)**					
0–20 d	820.45 ± 10.63	823.84 ± 7.50	825.37 ± 6.04	0.398	0.900
20–40 d	1220.94 ± 16.30	1225.95 ± 12.21	1225.69 ± 8.66	0.592	0.930
0–40 d	1020.69 ± 13.46	1024.89 ± 9.75	1025.53 ± 7.35	0.520	0.997
F/G	4.89 ± 0.88	5.05 ± 1.40	4.99 ± 1.19	0.956	0.391
**Apparent digestibility of male lambs (%)**					
Dry matter	67.90 ± 0.02 ^a^	65.48 ± 0.02 ^ab^	61.51 ± 0.02 ^b^	0.004	/
Crude protein	72.50 ± 0.94 ^b^	74.13 ± 1.43 ^ab^	75.24 ± 0.77 ^a^	0.018	/
Ether extract	62.28 ± 0.95 ^b^	63.42 ± 1.13 ^ab^	64.72 ± 1.02 ^a^	0.027	/
Neutral detergent fiber	44.03 ± 0.62	45.18 ± 0.98	46.63 ± 2.45	0.115	/
Acid detergent fiber	45.54 ± 0.01 ^a^	42.69 ± 0.01 ^b^	39.54 ± 0.01 ^c^	<0.01	/

^1^ Defective jujube was supplied to the daily diets at levels of 0%, CON; 15%, DJ15; 30%, DJ30; * Letters ‘a’, ‘b’, and ‘c’ within the same row denote significant differences among groups (*p* < 0.05). Unlabeled values indicate no statistical significance (*p* > 0.05).

**Table 5:** Slaughter performance and organ indices of lambs in three groups.

Items		Treatment ^1^		*p*-Value	
	CON	DJ15	DJ30	Treatment	Gender
Body weight before slaughter (kg)	30.74 ± 7.17	30.18 ± 4.51	32.55 ± 2.79	0.554	0.880
Hot carcass weight (kg)	13.73 ± 2.47	13.78 ± 1.94	14.13 ± 1.82	0.857	0.617
Dressing percentage (%)	45.09 ± 3.42	45.67 ± 2.02	43.45 ± 2.25	0.177	0.072
LD muscle area (cm^2^)	10.11 ± 1.65	11.44 ± 1.70	12.05 ± 2.14	0.074	0.475
Abdominal fat index (%)	1.15 ± 0.71	1.18 ± 0.58	0.93 ± 0.63	0.680	0.452
Mesenteric fat index (%)	0.60 ± 0.11	0.66 ± 0.16	0.68 ± 0.19	0.425	0.303
**Organ Index (%)**					
Heart	0.55 ± 0.07	0.57 ± 0.07	0.55 ± 0.04	0.651	0.072
Liver	1.54 ± 0.15	1.60 ± 0.19	1.66 ± 0.17	0.247	0.460
Spleen	0.29 ± 0.07	0.32 ± 0.06	0.31 ± 0.07	0.476	0.167
Lung	1.22 ± 0.08	1.37 ± 0.21	1.27 ± 0.21	0.135	0.895
Kidney	0.75 ± 0.30	0.68 ± 0.17	0.70 ± 0.33	0.836	0.123
Pancreas	0.13 ± 0.02	0.15 ± 0.03	0.15 ± 0.04	0.118	0.771

^1^ Defective jujube was supplied to the daily diets at levels of 0%, CON; 15%, DJ15; 30%, DJ30.

**Table 6:** Physical properties of the LD muscle of lambs in three groups.

Items *		Treatment ^1^		*p*-Value	
	CON	DJ15	DJ30	Treatment	Gender
pH _45 min_	6.36 ± 0.24	6.36 ± 0.39	6.32 ± 0.29	0.906	0.683
pH _24 h_	5.72 ± 0.09	5.77 ± 0.16	5.75 ± 0.14	0.729	0.208
L* _45 min_	28.87 ± 1.90	29.42 ± 2.00	28.67 ± 2.64	0.770	0.911
L* _24 h_	34.25 ± 2.69	34.49 ± 2.43	34.55 ± 2.97	0.981	0.770
a* _45 min_	11.25 ± 1.16	11.93 ± 2.31	11.90 ± 1.50	0.621	0.101
a* _24 h_	13.29 ± 0.97	13.11 ± 0.88	13.16 ± 0.90	0.837	0.598
b* _45 min_	3.64 ± 0.47	3.51 ± 0.63	3.76 ± 0.78	0.717	0.293
b* _24 h_	5.39 ± 1.16	5.32 ± 0.83	5.17 ± 1.16	0.946	0.147
Warner-bratzler shear force (N)	71.33 ± 4.35	69.68 ± 5.17	64.94 ± 7.98	0.065	0.783
Cooked meat percentage (%)	80.54 ± 0.06	81.26 ± 0.05	84.35 ± 0.05	0.158	0.808

^1^ Defective jujube was supplied to the daily diets at levels of 0%, CON; 15%, DJ15; 30%, DJ30; * L*, a* and b* are the core color difference indexes of CIE Lab color space: ‘L*’ is the brightness value; ‘a*’ is the redness value; ‘b*’ is the yellowness value. Measurements were performed at two specific time points: the angular scale ‘45 min’ represents the initial meat color measured at 45 min after slaughter, and the angular scale ‘24 h’ represents the meat color measured after the sample was refrigerated at 4 °C for 24 h.

**Table 7:** Basic chemical composition of the LD muscle of lambs in three groups.

Items		Treatment ^1^		*p*-Value	
	CON	DJ15	DJ30	Treatment	Gender
Moisture	73.57 ± 0.02	74.31 ± 0.02	75.12 ± 0.02	0.144	0.074
Ether extract	2.21 ± 0.01	2.50 ± 0.01	2.51 ± 0.01	0.682	0.900
Crude protein	20.70 ± 0.02	19.72 ± 0.02	19.53 ± 0.01	0.117	0.087

^1^ Defective jujube was supplied to the daily diets at levels of 0%, CON; 15%, DJ15; 30%, DJ30.

**Table 8:** Fatty acid composition of the LD muscle of lambs in three groups.

		Treatment ^1^		*p*-Value	
Fatty Acids (g/100 g Fatty Acid Methyl Esters) *	CON	DJ15	DJ30	Treatment	Gender
**Saturated**					
C8:0	0.12 ± 0.03	0.11 ± 0.05	0.12 ± 0.05	0.800	0.795
C10:0	0.18 ± 0.03	0.21 ± 0.07	0.18 ± 0.04	0.235	0.103
C11:0	0.14 ± 0.09	0.16 ± 0.10	0.13 ± 0.07	0.754	0.537
C12:0	0.15 ± 0.05	0.19 ± 0.09	0.17 ± 0.03	0.423	0.325
C14:0	2.41 ± 0.54	2.54 ± 0.87	2.57 ± 0.46	0.764	0.416
C15:0	0.43 ± 0.17	0.44 ± 0.13	0.43 ± 0.08	0.946	0.251
C16:0	26.13 ± 2.91	26.66 ± 4.10	26.24 ± 1.87	0.958	0.614
C17:0	0.83 ± 0.07 ^b^	0.95 ± 0.13 ^ab^	1.06 ± 0.20 ^a^	0.004	0.332
C18:0	19.80 ± 2.76	19.74 ± 1.48	20.62 ± 1.70	0.512	0.702
C20:0	0.12 ± 0.02	0.11 ± 0.03	0.10 ± 0.02	0.488	0.491
Total saturated fatty acids	50.03 ± 4.97	50.82 ± 4.98	51.54 ± 2.02	0.753	0.731
**Monounsaturated**					
C16:1	1.02 ± 0.14	1.17 ± 0.18	1.10 ± 0.19	0.092	0.295
C18:1N9T	0.33 ± 0.05	0.35 ± 0.04	0.33 ± 0.06	0.472	0.716
C18:1N9C	33.51 ± 3.94	34.86 ± 3.30	33.28 ± 3.91	0.306	0.839
C20:1	0.11 ± 0.02	0.12 ± 0.02	0.11 ± 0.02	0.578	0.870
**Polyunsaturated**					
C18:2N6C	8.49 ± 2.05	7.83 ± 3.15	8.55 ± 2.68	0.712	0.905
C18:3N3	0.27 ± 0.05	0.24 ± 0.06	0.26 ± 0.04	0.403	0.900
C20:2	0.11 ± 0.03	0.09 ± 0.02	0.09 ± 0.02	0.720	0.726
C20:3N6	0.31 ± 0.16	0.31 ± 0.15	0.27 ± 0.11	0.263	0.565
C20:4N6	4.08 ± 1.58	3.95 ± 2.08	4.08 ± 1.43	0.968	0.873
Total unsaturated fatty acids	47.98 ± 4.50	48.68 ± 4.70	47.97 ± 1.86	0.716	0.769

^1^ Defective jujube was supplied to the daily diets at levels of 0%, CON; 15%, DJ15; 30%, DJ30; * Letters ‘a’ and ‘b’ within the same row denote significant differences among groups (*p* < 0.05). Unlabeled values indicate no statistical significance (*p* > 0.05).

**Table 9:** Content of *Alternaria* toxins in diets of three treatment groups.

Concentrations (μg/kg)		Treatment ^1^	
	CON	DJ15	DJ30
Tenuazonic acid	56.6	481.9	1088.6
Alternariol	ND *	ND	ND
Alternariol monomethyl ether	ND	8.1	29.9
*Alternaria* tenuissima toxin	ND	ND	ND
Tentoxin	ND	ND	ND

^1^ Defective jujube was supplied to the daily diets at levels of 0%, CON; 15%, DJ15; 30%, DJ30; * ‘ND’ means not detected.

## Data Availability

The original contributions presented in this study are included in the article. Further inquiries can be directed to the corresponding authors.

## Abbreviations

The following abbreviations are used in this manuscript:

DJ	Defective jujube
CP	Crude protein
EE	Ether extract
DE	Digestible energy
TeA	Tenuazonic acid
AOH	Alternariol
AME	Alternariol methyl ether
NDF	Neutral detergent fiber
ADG	Average daily gain
ADFI	Average daily feed intake
F/G	Feed-to-gain ratio
DM	Dry matter
AIA	Acid insoluble ash
ALT	Alanine aminotransaminase
AST	Aspartate aminotransaminase
TP	Total protein
BUN	Blood urea nitrogen
GLU	Glucose
TG	Triglyceride
TC	Total cholesterol
HDL	High-density lipoprotein
LDL	Low-density lipoprotein
CAT	Catalase
SOD	Superoxide dismutase
GSH-Px	Glutathione peroxidase
MDA	Malondialdehyde
INS	Insulin
GH	Growth hormone
IGF-1	Insulin-like growth factor-1
BWS	Body weights before slaughter
LD	Longissimus dorsi
HCW	Hot carcass weight
WBSF	Warner-Bratzler shear force–Bratzler shear force
CMP	Cooked meat percentage
ATT	*Alternaria* tenuissima toxin
TEN	Tentoxin
FAs	Faty acids
MUFAs	Monounsaturated fatty acids
SFAs	Saturated fatty acids
UFAs	Unsaturated fatty acids
PUFAs	Polyunsaturated fatty acids
DMI	Dry matter intake
RCT	Reverse cholesterol transport
CYP 450	Cytochrome P450
